# Identification of Biomarkers for Footpad Dermatitis Development and Wound Healing

**DOI:** 10.3389/fcimb.2016.00026

**Published:** 2016-03-04

**Authors:** Juxing Chen, Guillermo Tellez, Jeffery Escobar

**Affiliations:** ^1^Research and Development, Novus International Inc.St. Charles, MO, USA; ^2^Department of Poultry Science, University of ArkansasFayetteville, AR, USA

**Keywords:** footpad dermatitis, lesion development, wound healing, gene expression, biomarker

## Abstract

Footpad dermatitis (FPD) is a type of skin inflammation that causes necrotic lesions on the plantar surface of the footpads in commercial poultry, with significant animal welfare, and economic implications. To identify biomarkers for FPD development and wound healing, a battery cage trial was conducted in which a paper sheet was put on the bottom of cages to hold feces to induce FPD of broilers. Day-of-hatch Ross 308 male broiler chicks were fed a corn–soybean meal diet and assigned to 3 treatments with 8 cages per treatment and 11 birds per cage. Cages without paper sheets were used as a negative control (NEG). Cages with paper sheets during the entire growth period (d 0–30) were used as a positive control (POS) to continually induce FPD. Cages with paper sheets during d 0–13 and without paper sheets during d 14–30 were used to examine the dynamic of FPD development and lesion wound healing (LWH). Footpad lesions were scored to grade (G) 1–5 with no lesion in G1 and most severe lesion in G5. Covering with paper sheets in POS and LWH induced 99% incidence of G3 footpads on d 13. Removing paper sheets from LWH healed footpad lesions by d 30. One representative bird, with lesions most close to pen average lesion score, was chosen to collect footpad skin samples for biomarker analysis. Total collagen protein and mRNA levels of tenascin X (TNX), type I α1 collagen (COL1A1), type III α1 collagen (COL3A1), tissue inhibitor of metalloproteinase 3 (TIMP3), and integrin α1 (ITGA1) mRNA levels were decreased (*P* < 0.05), while mRNA levels of tenascin C (TNC), tumor necrosis factor (TNF) α, Toll-like receptor **(**TLR) 4 and vascular endothelial growth factor (VEGF), IL-1β, and the ratio of MMP2 to all TIMP were increased (*P* < 0.03) in G3 footpads in POS and LWH compared to G1 footpads in NEG on d 14. These parameters continued to worsen with development of more severe lesions in POS. After paper sheets were removed (i.e., LWH), levels of these parameters gradually or rapidly returned to levels measured in NEG. Regression analysis indicated significant quadratic changes of these parameters to footpad lesion scores. In summary, these biomarkers were interrelated with dynamic changes of footpad lesion scores, suggesting they may be used as potential biomarkers for footpad lesion development and wound healing process.

## Introduction

Footpad dermatitis (FPD) is a type of skin inflammation that causes necrotic lesions on the plantar surface of footpads in broilers and turkeys (Shepherd and Fairchild, 2010). Chicken feet or paws are considered a culinary delicacy in many cultures and have become the third most important economic part of the chicken (chicken breast and wings are the first and second, respectively), with chicken paws accounting for approximately $280 million a year (US Poultry and Egg Export Council, 2009). In addition to causing downgrade of paw quality and economic loss, the occurrence of footpad lesions is now used as an audit criterion in welfare assessments of poultry production systems in Europe and U.S. (Martland, 1985; Mayne, 2005). Footpad lesions are also a source of microorganisms that can enter the blood stream and cause systemic infection of broilers and turkeys, so they are also a food safety issue (Martland, 1985; Campo et al., 2005; Manangi et al., 2012). FPD is characterized by inflammation and necrotic lesions, from superficial to deep, on the plantar surface of footpads and toes (Mayne, 2005). FPD is a multifactorial symptom that can be caused by many factors, such as diet, nutritional deficiencies, wet litter, litter material types, genetic strain, sex, bird weight, stock density, season, and management (Harms et al., 1977; Martland, 1985; Nagaraj et al., 2007). Among those factors, wet litter has been reported to be the most dominant predisposing factor to cause FPD in poultry (Martland, 1984; Wang et al., 1998; Mayne et al., 2007). Replacement of wet litter with dry litter recovered the footpad lesions in about 2 weeks (Nagaraj et al., 2007; Taira et al., 2014). However, in commercial poultry, sometimes it is not practical or economically likely to frequently replace litter material; therefore, alternative practices should be considered. Trace minerals such as Zn, Cu, and Mn are known to play a role in maintaining the structural integrity of different tissues, such as skin (Iwata et al., 1999; Marincola, 2003; Berger et al., 2007; Lansdown et al., 2007; Manangi et al., 2012; Vieira et al., 2013). Feed additives, such as enzymes, that improve digestion and help to maintain a stable intestinal microbiota and heathy gut wall barrier could improve fecal consistency and litter quality, therefore reducing footpad lesions (Van Der Aa and Additives, 2008). Nevertheless, there are no current tools or biomarkers that can be used to quantitatively measure the development and wound healing of FPD. Although the sequence of events leading to wound repair has been described at the cellular and, to a limited extent, at the protein level, this complex process is not completely interpreted. Several studies in mammal models have shown that genome transcriptional analysis tools promise to further understand the progression of events that take place during wound healing (Martin, 1997; Marincola, 2003; Gill and Parks, 2008; Kaczorowski et al., 2008). The primary goal of this study was to develop a model to induce high-incidence of moderate footpad lesions and to identify biomarkers for footpad lesion development and wound healing, which could help to provide strategies to prevent and intervene FPD and promote wound healing in commercial poultry.

## Materials and methods

### Animals and diets

A total of 264 hatchling Ross 308 male broiler chickens were randomly assigned to 3 groups, housed in battery cages with 11 chicks per cage, in environmentally controlled rooms. Cages measure about 61 × 71 cm and provide a floor area per bird of about 394 and 619 cm^2^ at the start and end of the study, respectively. Temperature was maintained at 34°C for the first 5 d and then gradually reduced according to normal management practices, until a temperature of 23°C was achieved. Hours of light (L) and dark (D) were provided as follows: d 1–3, 23L:1D; d 4–12, 18L:6 D; d 13–30, 16L: 8D. Birds were fed crumbled starter diets from d 0 to 12, switched to pelleted grower diet from d 12 to 26, and pelleted finisher diet from d 26 to 30 (Table 1). Birds had free access to feed and water at all times. Experimental diets were formulated to approximate nutritional requirements of broiler chickens (National Research Council, 1994). All research procedures were reviewed and approved by a licensed veterinarian. All studies performed by Novus International, Inc. are in accordance to the standards of the Guide for the Care and Use of Agricultural Animals in Research and Teaching (FASS, 2010).

**Table 1:** **Diet formulation and nutrient composition**.

**Ingredient**	**Starter, %**	**Grower,%**	**Finisher, %**
Corn	57.422	58.093	63.214
Soybean meal, 47.5% CP	35.813	34.165	29.517
Soybean oil	1.896	3.663	3.61
L-lysineHCl	0.221	0.061	0.046
MHA®^a^	0.298	0.2	0.154
L-threonine	0.06	- - -	- - -
L-tryptophan	0.166	0.146	- - -
Dicalcium phosphate 18.5%	1.283	1.037	0.928
Limestone	1.622	1.409	1.392
Salt	0.301	0.251	0.402
Choline chloride 60%	0.267	0.25	0.233
Mineral premix^b^	0.201	0.2	0.2
Vitamin premix^c^	0.101	0.1	0.1
Santoquin® Mixture 6^a^	0.02	0.02	0.02
Myco CURB®^d^	0.05	0.05	0.05
Coban® 90^e^	0.05	0.05	0.05
BMD® 60^f^	0.03	0.03	0.03
Sodium bicarbonate	0.144	0.22	- - -
Phyzyme® XP TPT^g^	0.005	0.005	0.005
Cibenza® DP100^a^	0.05	0.05	0.05
**CALCULATED NUTRIENTS**			
ME, kcal/kg	3025	3150	3200
Crude Protein %	22	21	19
Ca %	1.05	0.9	0.85
Total P, %	0.62	0.57	0.53
Available P, %	0.5	0.45	0.42

a *MHA, Santoquin, and Cibenza are registered marks of Novus International, Inc., St. Charles, MO*.

b *Vitamin premix supplied per kilogram of diet: retinol, 9.2 mg; cholecalciferol, 100 μg; dl-α-tocopherol, 90 mg; menadione, 6 mg; thiamine, 6.2 mg; riboflavin, 26.5 mg; pantothenic acid, 39.7 mg; niacin, 100 mg; pyridoxine, 11 mg; folic acid, 4 mg; biotin, 0.3 mg; cyanocobalamin, 0.1 mg*.

c *Mineral premix supplied per kilogram of diet: Mn, 64 mg; Zn, 64 mg; Fe, 37 mg; Cu, 16 mg; I, 0.7 mg; Se, 0.3 mg*.

d *Myco CURB is a registered mark of Kemin Industries, Inc., Des Moines, IA*.

e *Coban is a registered mark of Elanco Animal Health, Indianapolis, IN*.

f *BMD is a registered mark of Zoetis, Inc., Florham Park, NJ*.

g *Phyzyme is a registered mark of Danisco Animal Nutrition, Marlborough, UK*.

### Experimental design

To identify biomarkers for FPD development and wound healing, a battery cage trial was conducted in which an absorbent paper sheet was put on the bottom of cages to hold feces and induce FPD of broilers. Papers used have a polyethylene lining on the bottom (Benchkote®, GE Healthcare Life Sciences, Pittsburgh, PA) to provide accumulation of excreta and moisture; papers were not changed during the study unless otherwise indicated. Chickens were assigned to 3 treatments with 8 cages per treatment and 11 birds per cage. Cages without paper sheets were used as a negative control (NEG). Cages with paper sheets during the entire growth period (d 0–30) were used as a positive control (POS) to continually induce FPD. Cages with a paper sheet during d 0–13 and without a paper sheet during d 14–30 were used to examine the dynamic of FPD development and lesion wound healing (LWH). At 12 and 30 d, body weight, body weight gain, and feed intake were recorded in each cage to calculate feed conversion.

### Lesion score and sample collection

At 13, 21, 26, and 29 d-of-age, right and left chicken paws from all birds were scored for presence and severity of FPD gross lesions by the same scorer throughout the study. Score was performed by modification of visual ranking on a scale of 1–5 (1, no lesions; 2, lesions of 2 mm or less; 3, lesions of 2 to 7 mm; 4, lesions larger than 7 mm; 5, lesions larger than 7 mm, including toe lesions) (Nagaraj et al., 2007). This scoring was accomplished by first dipping the feet in a bucket of water and removing all adhering material from the foot pad. At 14, 22, 27, and 30 d-of-age, one representative chicken from each pen with lesions most close to pen average lesion score was chosen to collect footpad skin samples for total collagen protein and gene expression analysis as describe below.

### Total collagen analysis

One piece of the left footpad of each bird per pen was collected and flash frozen on dry ice for collagen analysis. Hydroxyproline (Hyp) plays a key role for collagen stability and is present in only a few proteins, such as collagen and elastin. Therefore, Hyp has been used for many years as a marker to determine the amount of collagen present in a tissue. Footpad skin Hyp content was analyzed to estimate collagen content (Ignat'eva et al., 2007) with some modifications. For the assay, about 100 mg of tissue was homogenized in 6 ml of 6N HCl in a glass tube using a Cole Parmer Lab Gen 700 (Cole-Parmer North America, Vernon Hills, IL) for about 30 s, and hydrolyzed at 110°C for 24 h after adding an additional 6 ml of HCl. One milliliter of hydrolyzed sample was neutralized with 2 ml of 3N NaOH and diluted 2 × with distilled H_2_O. Serial dilutions of 0.1 mg/ml trans-4-hydroxy-L-proline were used as standard to generate standard curve. A 0.25-ml aliquot of 0.01 M CuSO_4_, 0.25 ml 2.5 N of NaOH, and 0.25 ml of 6% H_2_O_2_ were added in succession into glass tubes with 0.5 ml of diluted samples or standards. After vortex for 1 min, tubes were incubated in water bath at 80°C for 5 min with frequent shaking. After cooling tubes on ice, 1 ml of 3N H_2_SO_4_ and 0.5 ml of 4-dimethyl-amino-benzaldehyde were added in succession into glass tubes followed by incubation in water bath at 70°C for 16 min with slight shaking. Supernatants was transferred into cuvettes and read at 560 nm. Hyp content per gram of tissue was determined according to a standard curve. Total collagen content was calculated by multiplication of the Hyp content by 7.5 (Bonifer and Froning, 1996; Ignat'eva et al., 2007).

### Quantitative reverse-transcription polymerase chain reaction

One piece of each of the left footpad skin samples (stratum corneum, epidermis, dermis, and a very thin layer of subcutaneous tissue) was stored in RNAlater® (Life Technologies, Grand Island, NY) at 4°C for 24 h and then at −20°C until total RNA isolation. Total RNA was isolated from footpad skin samples using MagMAX™-96 for Microarrays Kit (Life Technologies, Grand Island, NY) after homogenization in Trizol® (Life Technologies, Grand Island, NY). One microgram of total RNA, 11-mer oligo mix from Fluoresentric, and M-MLV Reverse Transcriptase (Life Technologies, Grand Island, NY) were used to synthesize cDNA according to the manufacturers' instructions. Primers used for qRT-PCR are described in Table 2. Levels of mRNA were measured by quantitative PCR using Applied Biosystems® SYBR® Green PCR Master Mix (Life Technologies, Grand Island, NY) and a 7500 Fast Real-Time PCR System. Results were expressed as the level relative to the corresponding housekeeping gene *actin*. All primers were verified for the efficiency (100 ± 10%) and linearity (*r*^2^ ≥ 0.99) of amplification.

**Table 2:** **List of primers used for qRT-PCR**.

**Gene**	**Forward primer**	**Reverse primer**
TIMP2	GGATTCAGGGAATGACATTTATGG	ATCTTGATCTGCTTCACTTCGTACTG
TIMP3	CTCCAACTTCGGCCACTCA	CTTCCACCCTCTGGATGCA
TIMP4	TCATCTGCGATTCTGCTTTAGTG	GGCAGGAACCACCTTTTCAC
MMP2	GGACTGTAAACCCTCGAGGAAA	CAGATCAGGCCAGAATGTAGCA
VEGF	AGAAAGGCCGGTACAAACCA	AGTGCTTTCTCCTCTCTGAGCAA
TNX	AGCCCCTTGAGACCACCTTT	TGCTGAAGGACAGAGCAGTGTAG
TNC	GCTCTCAAATTTCTCCTCCAGTCT	CCTTTTCAAAGCTGATGGAGTCTT
COL1A2	GCAGAATACTACCGGGCTGATC	TTTTCAGAGTGGCATCAACTTCA
COL3A1	ATGTGAAGGCTGGCTCAGTTG	GTCCCGGAAAGCCACTAATG
ITGA2	CCCGAATCTGGAACAGTACCTT	TGCCTCAGCAAAGAGTTGCA
ITGB1	GCAAGGTGGAAGTGACTGCAT	CTGCCTGTATACATTCCCCACAT
actin	CAACACAGTGCTGTCTGGTGGTA	ATCGTACTCCTGCTTGCTGATCC
TNFα	TGTTCTATGACCGCCCAGTTC	GACGTGTCACGATCATCTGGTT
TLR4	AGTCTGAAATTGCTGAGCTCAAAT	GCGACGTTAAGCCATGGAAG

### Statistical analyses

All data were tested for normality and subjected to analysis of variance as a completely randomized design using the proc mixed procedure of SAS 9.4 (SAS Institute Inc., 2002, Cary, NC). Each cage was used as the experimental unit for the analysis. Growth performance including BW, BWG, and FCR used the average data per cage. qRT-PCR used individual measurement from one representative bird per cage. Data are expressed as mean ± standard error (SE). Statistical differences among means were determined by using Duncan's multiple-range test.

## Results

Growth performance parameters among NEG, POS, and LWH broiler chickens at 12 and 30 d-of-age are summarized in Table 3. At 12 d-of-age, no differences were determined among experimental groups (*P* > 0.30); however, at the end of the trial at d 30, a reduction (*P* < 0.03) in body weight, body weight gain, and feed conversion was determined in POS, compared with NEG and LWH groups, suggesting that direct contact of chicken feet with excreta compromised growth performance. In the present study, no differences (*P* > 0.14) in cumulative mortality were determined among groups at 12 or 30 d (Table 3).

**Table 3:** **Growth performance parameters among negative (NEG) and positive (POS) controls, and development and lesion wound healing (LWH) in broiler chickens at different ages**.

**Item**	**Treatment**			**SEM**
	**NEG**	**POS**	**LWH**	
**12 d of age**				
Body weight, kg	0.36	0.36	0.35	0.01
Body weight gain, kg	0.31	0.31	0.31	0.01
Feed conversion ratio, kg/kg	1.15	1.16	1.16	0.01
Mortality, %	4.02	0.18	2.04	1.81
**30 d of age**				
Body weight, kg	1.60^a^	1.54^b^	1.65^a^	0.02
Body weight gain, kg	1.55^a^	1.49^b^	1.61^a^	0.02
Feed conversion ratio, kg/kg	1.37^b^	1.46^a^	1.37^b^	0.01
Mortality, %	5.51	0.34	2.88	1.78

*Cages without paper were used as a NEG. Cages with papers during the entire growth period (d 0–30) were used as a POS to continually induce footpad dermatitis. Cages with paper during d 0–13 and without paper during d 14–30 were used to examine the dynamic of FPD development and wound healing (LWH)*.

a, b *Means within a row with different superscripts differ at P < 0.05*.

*Reported SEM is the average SEM of the individual least-square means*.

**Figure 1:**
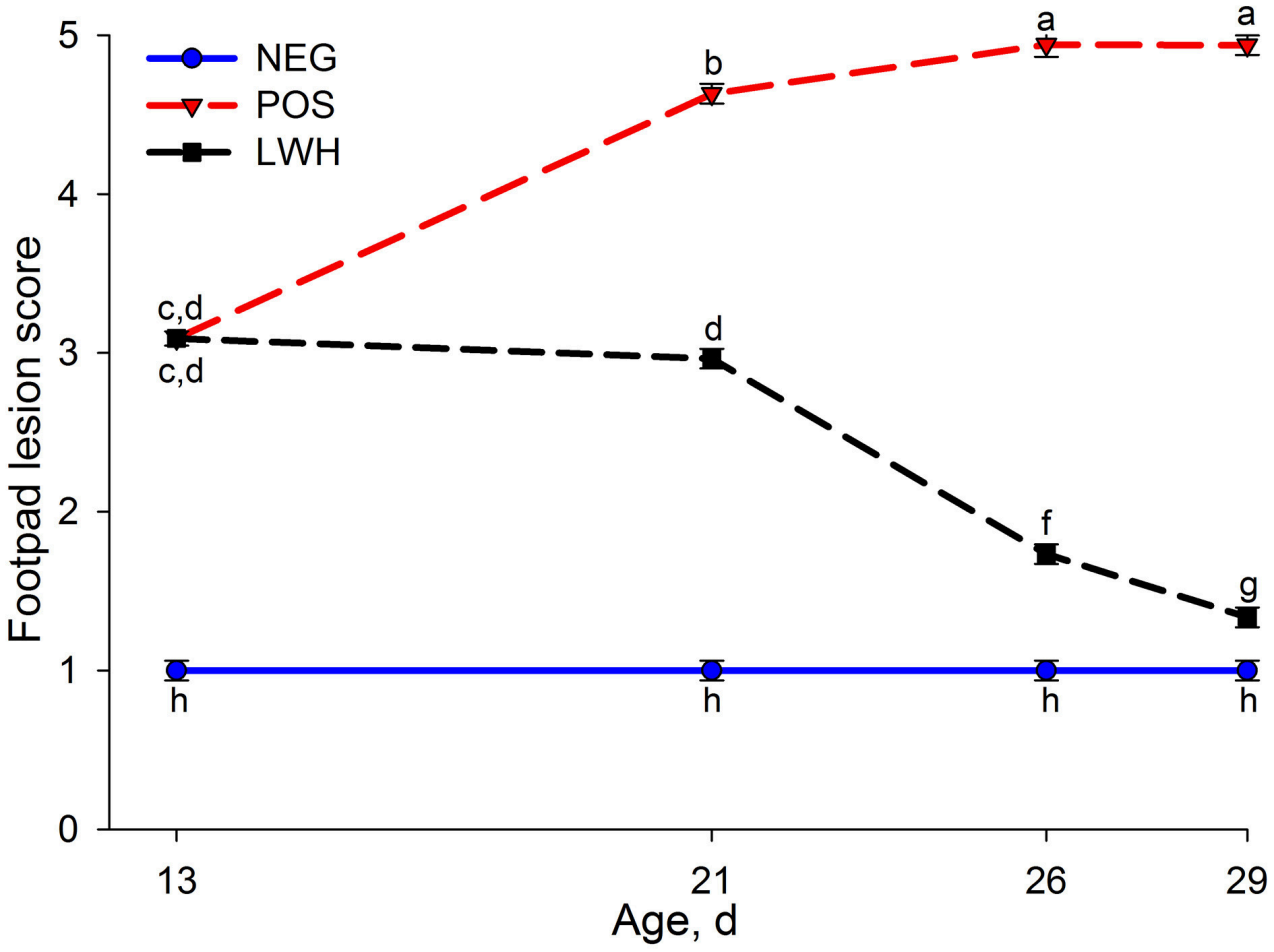
**Footpad lesion score among negative (NEG) and positive (POS) controls, and development and lesion wound healing (LWH) in broiler chickens at different ages**. Means with different letters differ at *P* < 0.05.

Figure 1 shows the footpad lesion score in NEG, POS, and LWH broiler chickens at 13, 21, 26, and 29 d-of-age. Covering paper sheet in POS and LWH induced 99% incidence of G3 footpad lesions on d 13, compared with NEG (i.e., cages without paper sheet), which remains without lesions throughout the study. After removing the paper sheet at d 13 in cages of the LWH group, footpad lesions showed progressive recovery, and by d 21 this segregation was different (*P* < 0.0001) from birds in POS group (Figure 1).

**Figure 2:**
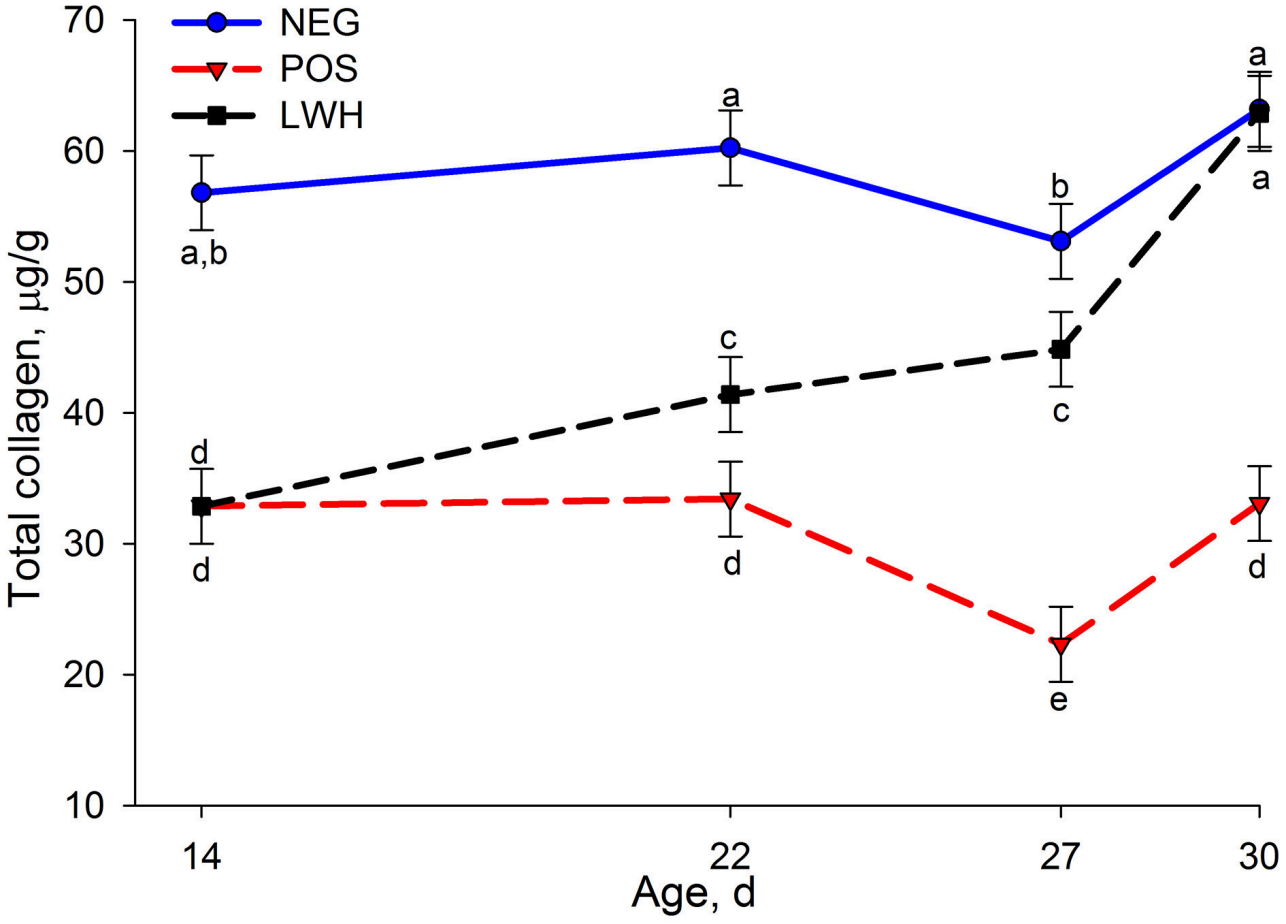
**Total collagen protein levels in negative (NEG) and positive (POS) controls, and in lesion wound healing (LWH) broiler chickens at different ages**. Means with different letters differ at *P* < 0.05.

Figure 2 shows results of total collagen among NEG, POS, and LWH broiler chickens at 14, 22, 27, and 30 d-of-age. Chickens in the NEG group without footpad lesions had the highest concentration of total collagen at each time point of evaluation. However, when birds in both groups (POS and LWH) with the paper sheet in cages developed footpad lesions, there was a reduction (*P* < 0.0001) of total collagen protein at d 14. Nevertheless, it was remarkable to visually quantify that with gradual healing of footpad lesions (Figure 1), the total collagen protein levels in LWH chickens gradually increased and were higher (*P* < 0.0001) than POS at d 27, and 30. By d 30, the concentration of total collagen in LWH was almost the same (*P* = 0.87) as that in healthy footpads in NEG group (Figure 2), indicating a full recovery of FPD.

**Figure 3:**
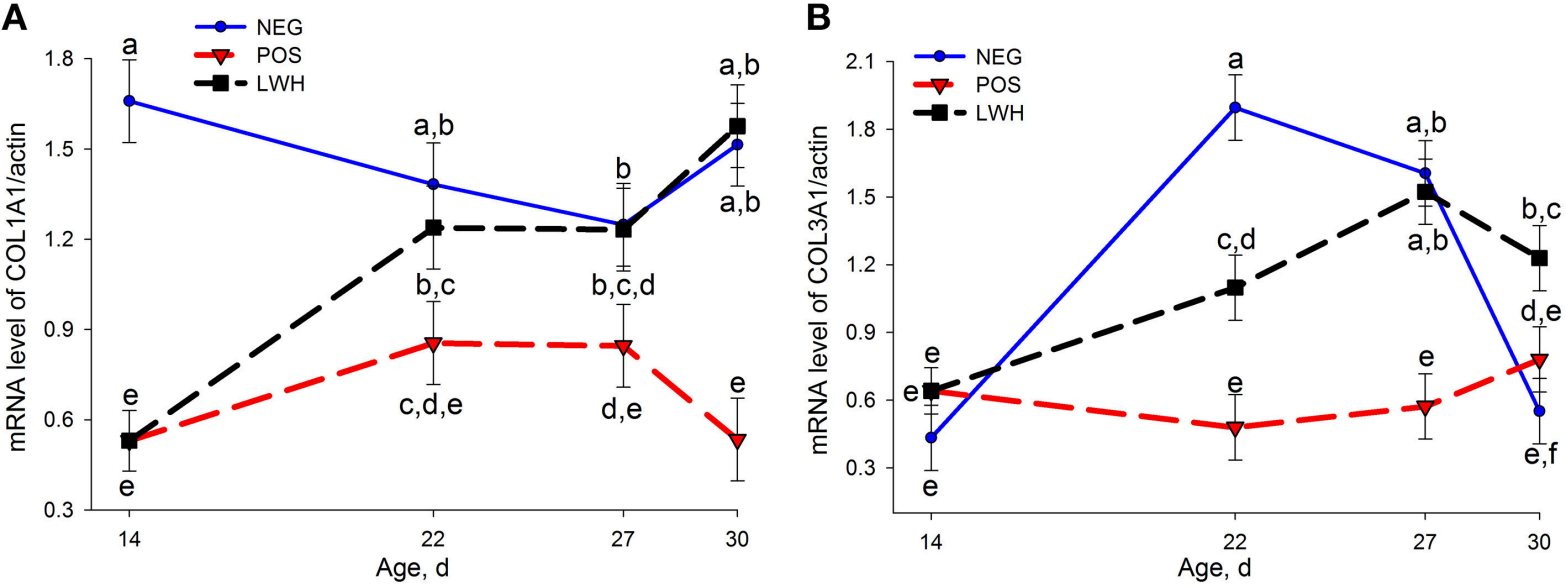
**Gene expression of COL1A1 (A) and COL3A1 (B) in negative (NEG) and positive (POS) controls, and in development and lesion wound healing (LWH) broiler chickens at different ages**. Means with different letters differ at *P* < 0.05.

**Figure 4:**
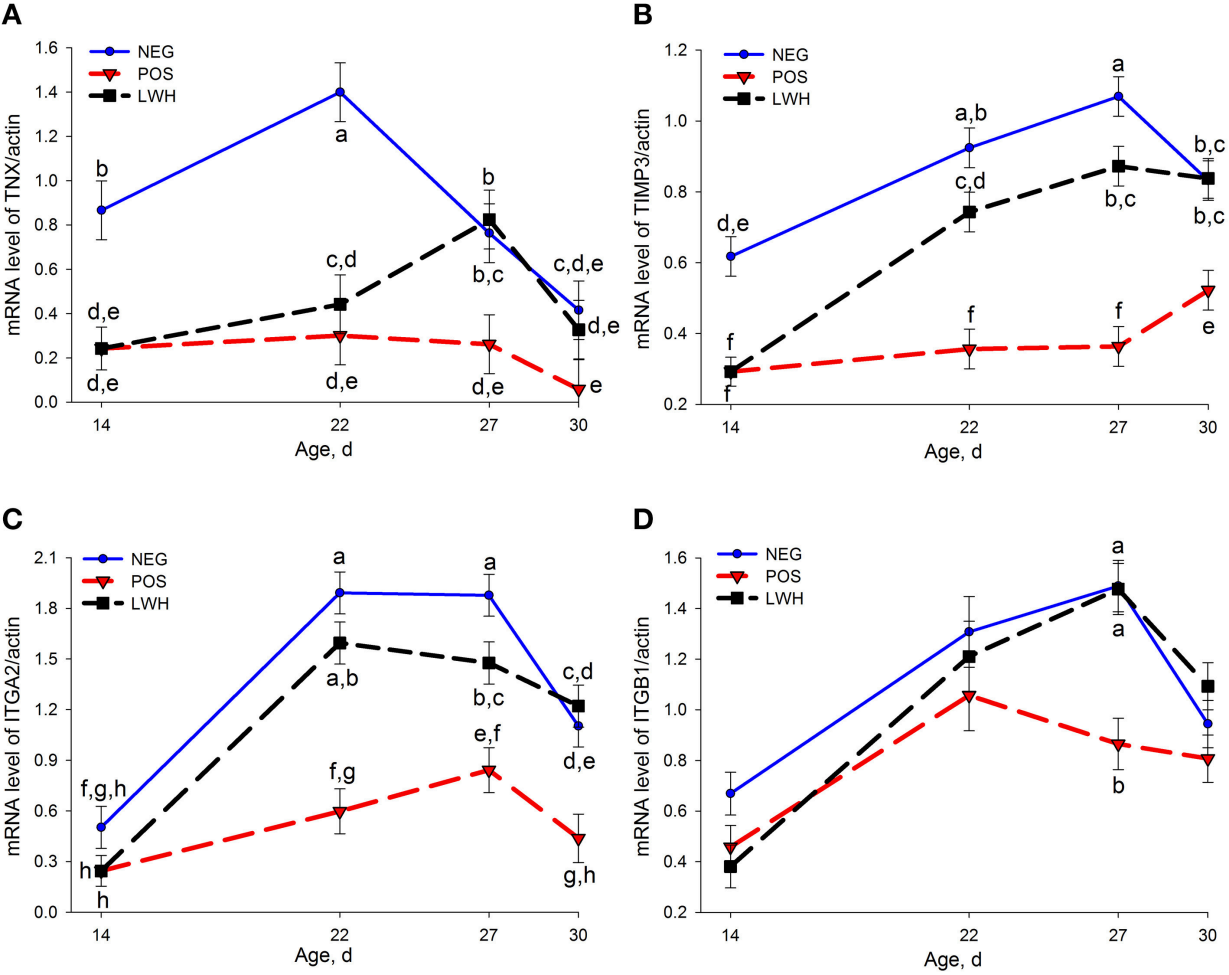
**Gene expression of TNX (A), TIMP3 (B), ITGA2 (C) and ITGB1 (D) in negative (NEG) and positive (POS) controls, and in development and lesion wound healing (LWH) broiler chickens at different ages**. There was no interaction between treatments and age for ITGB1 **(D)**, treatment differences in birds at same age were shown in **(D)**. Means with different letters differ at *P* < 0.05.

**Figure 5:**
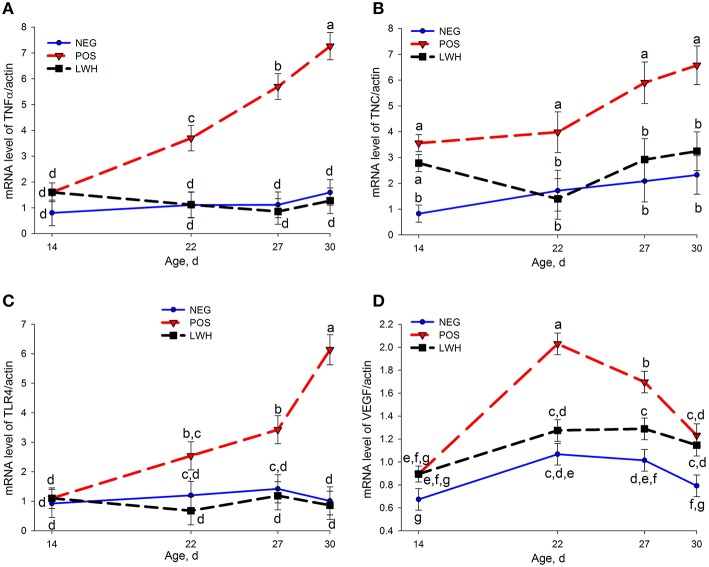
**Gene expression of TNFα (A), TNC (B), TLR4 (C), and VEGF (D) in negative (NEG) and positive (POS) controls, and in development and lesion wound healing (LWH) broiler chickens at different ages**. There was no interaction between treatments and age for TNC **(B)**, treatment differences in birds at same age were shown in **(B)**. Means with different letters differ at *P* < 0.05.

Out of 22 genes evaluated by qRT-PCR, 8 genes showed differential expression (*P* < 0.004) among treatments and the age of birds, 2 genes (TNC and ITGB1) showed marked differences (*P* < 0.03) among treatments within the same age of birds. The mRNA levels of tenascin X (TNX), type I alpha 1 collagen (COL1A1), type III alpha 1 collagen (COL3A1), tissue inhibitor of metalloproteinase 3 (TIMP3), integrin α2 (ITGA2), and integrin β1 (ITGB1) were reduced (*P* < 0.03) in POS group at almost all points of evaluation when compared with NEG (Figures 3, 4). Both groups with a paper sheet (POS and LWH) had similar levels of these biomarkers at d 14; however, after removing the paper sheets in the LWH group, their levels were gradually or rapidly increased to levels close to NEG group, indicating a recovery of FPD (Figures 3, 4). On the other hand, the mRNA levels of tenascin C (TNC, *P* < 0.0001), and tumor necrosis factor alpha (TNFα, *P* = 0.001) were increased in footpads with lesions in both POS and LWH groups, compared to healthy footpads in NEG on d 14 (Figure 5). After removing the paper sheet in LWH treatment, with recovery of footpad lesions (Figure 1), mRNA levels of TNC, TNFα, and TLR4 were reduced when compared with POS chickens at d 22 (*P* < 0.06), 27 (*P* < 0.02), and 30 (*P* < 0.002) (Figure 5). With development of more severe footpad lesions in POS treatment (Figure 1), the mRNA levels of TNC, TNFα, and TLR4 continued to increase over time and were markedly higher (*P* < 0.06) than both NEG and LWH (Figure 5). The mRNA levels of VEGF were increased (*P* < 0.07) in footpads with moderate lesions (POS and LWH treatments) at d 14, and markedly increased (*P* < 0.006) at d 22, 27, and 30 in footpad with severe lesions of POS compared with the healthy footpads of NEG (Figure 5). After the healing of footpad lesions, VEGF mRNA levels in LWH group were reduced (*P* < 0.003) compared to the POS group at d 22 and 27 (Figure 5).

The mRNA levels of IL-1β and MMP2 on d 14 and d 27 were also measured. IL-1β mRNA levels were markedly increased (*P* < 0.04) in footpads with moderate lesions (POS and LWH) on d 14 and remained at high levels in POS with continuous development of more severe lesions in POS. IL-1β mRNA levels decreased (*P* < 0.005) in LWH to levels comparable to those of healthy footpads (i.e., NEG) on d 27 (Table 4). The gene expression of MMP2 was not different (*P* > 0.44) among treatments on d 14 or d 27, but it increased (*P* < 0.0001) with age. Compared to healthy footpads in NEG, on d 14, ratios of MMP2 to TIMP3 and TIMP (TIMP2 + TIMP3 + TIMP4) were increased (*P* < 0.03) in footpads with moderate lesions (POS and LWH treatment); on d 27, ratios of MMP2 to TIMP2, TIMP3, TIMP4, and TIMP (TIMP2 + TIMP3 + TIMP4) were increased (*P* < 0.05) in footpads with severe lesions (POS). However, with healing of footpad lesions in LWH treatment, all ratios decreased to levels comparable (*P* > 0.16) to NEG treatment on d 27(Table 4).

**Table 4:** **Gene expression of TIMP2, TIMP3, TIMP4, MMP2, IL-1β, and the ratio of MMP2 to TIMP2, TIMP3, TIMP4, and TIMPs in negative (NEG) and positive (POS) controls, and development and lesion wound healing (LWH) in broiler chickens at different ages**.

**Item**	**Treatment**			**SEM**	***P*-value**
	**NEG**	**POS**	**LWH**		
**14 D OF AGE**					
TIMP2	0.742^a^	0.496^b^	0.496^b^	0.035	< 0.0001
TIMP3	0.618^a^	0.293^b^	0.293^b^	0.057	0.0006
TIMP4	0.607^a^	0.320^b^	0.320^b^	0.085	0.027
MMP2	0.548	0.452	0.452	0.086	0.441
MMP2/TIMP2	0.730	0.888	0.888	0.131	0.411
MMP2/TIMP3	0.827^b^	1.541^a^	1.541^a^	0.133	0.001
MMP2/TIMP4	0.894	2.213	2.213	0.657	0.175
MMP2/TIMP	0.258^b^	0.390^a^	0.390^a^	0.038	0.026
IL-1β	2.144^b^	76.486^a^	76.486^a^	23.024	0.035
**27 D OF AGE**					
TIMP2	1.005	0.956	1.072	0.082	0.61
TIMP3	1.069^a^	0.364^c^	0.873^b^	0.049	< 0.0001
TIMP4	4.053^a^	1.844^b^	2.691^b^	0.587	0.045
MMP2	1.776	1.928	1.840	0.178	0.839
MMP2/TIMP2	1.794^b^	2.174^a^	1.736^b^	0.123	0.048
MMP2/TIMP3	1.680^b^	5.504^a^	2.158^b^	0.611	0.0005
MMP2/TIMP4	0.477^b^	1.784^a^	1.171^b^	0.345	0.049
MMP2/TIMP	0.303^b^	0.735^a^	0.465^a,b^	0.093	0.014
IL-1β	1.337^b^	142.529^a^	2.333^b^	29.931	0.005

a, b, c *Means within a row with different superscripts differ at P < 0.05*.

Regression analysis using all data in three treatments was employed to determine the correlation of parameters measured in this study with footpad lesion scores. There were quadratic relationships (*P* < 0.03) among all genes shown in Table 5 and footpad lesion scores.

**Table 5:** **Correlation of total collagen protein and mRNA levels of COL1A1, COL3A1, TNFα, TLR4, IL-1β, TNC, VEGF, TNX, ITGA2, ITGB1, TIMP3, TIMP2, TIMP4, MMP2, MMP2/TIMP2, MMP2/TIMP3, MMP2/TIMP4, and MMP2/TIMPs with footpad lesion score**.

**Item**	**Quadratic effect**	
	***r*^2^**	***P*-value**
Total collagen	0.545	< 0.0001
COL1A1	0.322	< 0.0001
COL3A1	0.136	< 0.0001
TNFα	0.550	< 0.0001
TLR4	0.370	< 0.0001
IL-1β	0.308	0.0004
TNC	0.284	< 0.0001
VEGF	0.285	< 0.0001
TNX	0.245	< 0.0001
ITGA2	0.217	< 0.0001
ITGB1	0.127	0.002
TIMP3	0.456	< 0.0001
TIMP2	0.166	0.0002
TIMP4	0.117	0.0032
MMP2	0.232	0.0034
MMP2/TIMP2	0.263	0.0014
MMP2/TIMP3	0.556	< 0.0001
MMP2/TIMP4	0.151	0.0294
MMP2/TIMP	0.348	0.0001

## Discussion

FPD is characterized by inflammation and necrotic lesions, ranging from superficial to deep, on the plantar surface of footpads and toes. Deep ulcers may lead to abscesses and thickening of underlying tissues and structures (Martland, 1985; Shepherd and Fairchild, 2010). In the present study, at d 30, POS chickens had the worst performance compared to NEG and LWH (Table 3 and Figure 1). These results indicate that when birds were directly in contact with their excreta (paper sheet in the cage held the excreta), they could have been enterically challenged, behaviorally affected, or a combination of factors that led to poor growth performance. On d 13, 75–100% of birds developed footpad lesions in cages that had a paper sheet, indicating that direct contact with excreta and moisture accumulation was very effective to irritate footpad skin and induce footpad lesions in such a short period of time (13 d). Once the paper sheet was removed on d 14 in the LWH group, and there was no direct contact of footpad with excreta, footpad lesions started to heal with significant reduction of footpad lesion scores on d 21 and 26 when compared with POS group. By d 29 in LWH treatment, footpad lesions were almost completely healed with lesion score of 1.2, and performance parameters were recovered and similar to the NEG chickens. These results suggest that the paper insult used in this study induced footpad lesions and compromised growth performance in broilers.

Collagen is the main and most abundant structural protein in the extracellular space in various connective tissues, such as tendons, ligaments and skin in animals, making up to 35% of the whole-body protein content (Opsahl et al., 1982; Martin, 1997; Marincola, 2003). Birds grown in cages covered with a paper sheet had lower total collagen levels on d 14 than birds grown in cages without paper sheets. Once paper sheets were removed on d 14, a significant decrease of footpad lesion score (healing of lesions), and a gradual increase of collagen levels was observed on d 22 and 27, and by d 30 the total collagen levels increased to the same levels as birds grown without a paper sheet (NEG), suggesting that total collagen levels were associated with the footpad wound-healing process.

During the initial response to a cutaneous wound, powerful transcriptional activation of pro-inflammatory stimuli alert the host defense. This complex process requires the collective efforts of different cell lineages during phases of proliferation, migration, matrix synthesis, and contraction, as well as the growth factor and matrix signals present at a wound site (Chebassier et al., 2004). Subsequently, and in the absence of infection, inflammation subsides and is then replaced by angiogenesis and remodeling. Some of these cells, including immune cells, endothelial cells, keratinocytes, and fibroblasts, endure discernible changes in gene expression and phenotype (Xu et al., 2013). In mammalian species, genome-wide transcriptional analysis tools have helped to define this multifaceted progression of events (Martin, 1997; Marincola, 2003). In the present study, we focused on relative mRNA levels of 22 genes potentially involved in wound healing in mammalian species and found that 10 were associated with footpad lesion development and wound healing.

Consistent with the loss of total collagen protein in lesioned footpads and the recovery of total collagen protein in healing footpads, mRNA levels of COL1A1 but not COL3A1 were significantly decreased in footpads with moderate lesions on d 14, both COL1A1 and COL3A1 remained at lower levels in POS than NEG but were gradually increased in LWH on d 22 and 27 to similar levels as healthy footpads in NEG. On d 30, when footpad lesions were almost completely healed, COL1A1 mRNA levels were similar to NEG and higher than POS, but COL3A1 mRNA levels were higher than both NEG and POS, indicating that COL3A1 is not required in healthy footpads in later stages of footpad maturation and development, but highly demanded during the wound-healing process.

Integrins are heterodimeric glycoproteins that contain non-covalently associated α and β subunit. They are expressed in basal layer keratinocytes *in vivo* and responsible for intercellular or cell-substrate adhesion and sensing mechanical stress (Tuckwell et al., 1995; Tenaud et al., 1999; Boo and Dagnino, 2013). Integrin α2 binds types I, II, III, and XI collagen (Leask, 2013). Integrin β1 is an essential mechanosensory receptor in dermal fibroblasts, necessary for fibroblasts to maximally sense mechanical tension, modulate collagen deposition, and promote connective tissue homeostasis (Leask, 2013). Integrin α2 subunit forms a heterodimer with integrin β1 subunit and acts as a functional cellular receptor for type I collagen fibrils to facilitate both cell spreading on type I collagen matrix and contraction of type I collagen gel (Tuckwell et al., 1995; Jokinen et al., 2004). In the present study, the expression of integrin α2 and β1 was lower in footpads with moderate lesions in POS and LWH than NEG on d 14, and remained lower than NEG in footpads with severe lesions in POS on d 22, 27, and 30. After removing the paper sheet, with gradual healing of footpad lesions, the expression of integrin α2 and β1 rapidly increased to, and maintained at, similar levels as healthy footpads in NEG. The decrease of integrin α2 and β1 in lesioned footpads and increase of integrin α2 and β1 in healing footpads is consistent with reduction of COL1A in lesioned footpads and increase of COL3A1 in healing footpads. It suggests the loss of collagen content and extracellular matrix (ECM) adhesion during lesion development, restoration of collagen deposition, and cell-ECM adhesion during wound healing process. These findings further underlined the important role of integrin α2β1 in collagen deposition and organization.

Tenascins are ECM glycoproteins that are abundant in the ECM of various organs, such as mammary gland and hair follicles, of developing vertebrate embryos, absent or expressed at very low levels in adult mammary gland, and reappear around healing of wounds (Mackie et al., 1988; Seifert et al., 2012). These glycoproteins contribute to ECM structure and influence the physiology of cells in contact with the tenascin-containing environment (Chiquet-Ehrismann and Tucker, 2004). There are four members of the tenascin gene family: tenascin-C (TNC), tenascin-R, tenascin-X (TNX), and tenascin-W. TNX regulates structure and stability of elastic fibers and organizes collagen fibrils in the ECM, and is thought to function in matrix maturation during wound healing (Wu et al., 2014). Tenascin X deficiency in humans leads to a recessive form of Ehlers-Danlos Syndrome, an inherited connective tissue disorder characterized by hypermobile joints, fragile skin, and reduced dermal collagen content (Burch et al., 1997; Zweers et al., 2004; Lindor and Bristow, 2005).

TNX gene expression was significantly reduced in both POS and LWH groups on d 14 and 22, when compared to NEG group, and remained significantly lower in POS than NEG on d 27 and 30. These results suggest that significant reduction of TNX gene expression is an indication of necrosis occurring in the connective tissue of footpad skin. The study in TNX-null mice showed that TNX is not involved in matrix deposition in the early phase of wound healing, but is required in the later phase of matrix remodeling and maturation (Egging et al., 2007). Consistent with TNX-null mice study, after removing paper sheet on d 14 in LWH, TNX gene expression in healing footpads did not increase on d 22 but significantly increased on d 27 to the similar levels as healthy footpads in NEG. These results suggest TNX expression was not needed during early healing phase (d 22), but was required during the later matrix remodeling and maturation phase. Therefore, we can conclude that reduction of TNX gene expression is an indication of footpad lesion development, and restoration of TNX gene expression is an indication of late phase FPD wound healing (Figure 3). These findings further demonstrate the important role of TNX in structural integrity of connective tissue.

TNC gene expression is regulated by mechanical stress (Chiquet-Ehrismann and Tucker, 2004). It is highly expressed in connective tissue surrounding tumors, in wounds, and in inflamed tissues where it may regulate cell morphology, growth, and migration (Chiquet-Ehrismann and Tucker, 2004). TNC gene expression was up-regulated in lesioned skin compared to non-lesioned skin in patients with atopic dermatitis (Ogawa et al., 2005). In this study, consistent with the increase of TNC expression in lesioned skin in atopic dermatitis patients, we found TNC gene expression was dramatically increased in lesioned footpads, but was rapidly decreased to similar levels as in healthy footpads of NEG once lesions started to heal on d 22, which indicates upregulation of TNC gene expression is an indicator of footpad skin lesions and the downregulation of TNC gene expression is an indicator of the early phase of wound healing.

TIMP regulates cell migration in wound healing by modulating the activity of specific metalloproteinases (MMP) (Gill and Parks, 2008). The balance between MMP and TIMP is critical for the normal wound-healing process (Martins et al., 2013). A low MMP/TIMP ratio has been used as a good predictor of successful wound healing in diabetic foot ulcers (Muller et al., 2008). In diabetic patients, the expression of MMP-9, MMP-2, and MMP-8 were increased, while TIMP-2 was decreased, which creates an unfavorable ratio of MMP/TIMP (Uemura et al., 2001; Lobmann et al., 2002). The abnormally elevated level of MMP and reduced levels of TIMP may impair cell migration, and result in sustained inflammation with net increased tissue destruction. In chronic diabetic foot lesions, local administration of protease inhibitors reduces the ratio of MMP/TIMP and improves wound healing (Jokinen et al., 2004; Fadini et al., 2005). In the present study, TIMP3 gene expression was significantly decreased in POS on d 14 and remained low on d 22 and 27, when compared to NEG footpads. Interestingly, after removing the paper sheet on d 14 in LWH treatment, and as lesions started to heal, TIMP3 gene expression gradually increased, returning to the same levels as NEG chickens in healed footpads on d 30 (Figure 3). These results suggest that in footpad with lesions, the amount of TIMP3 was not sufficient to inhibit MMPs-mediated matrix degradation, and the degradation of ECM protein leads to necrotic skin lesions on footpads, during the wound healing period (i.e., after removing paper sheet) TIMP3 expression was increased to inhibit matrix protein degradation, therefore promoting the wound-healing process. In addition, the ratio of MMP2 to TIMP2, TIMP3, TIMP4, and TIMP (TIMP2 +TIMP3 +TIMP4) were numerically or significantly increased in footpads with moderate lesions (d 14) and footpads with severe lesions (d 30) compared to healthy footpads in NEG, but returned to normal levels as healthy footpads in healing footpads in LWH. These results suggest that TIMP3 and the ratio of MMP2 to TIMP could be used as a biomarker of footpad lesion development and wound healing.

Wound healing, whether initiated by trauma, microbes, or foreign materials, proceeds via an overlapping pattern of events including coagulation, inflammation, epithelialization, formation of granulation tissue, and matrix and tissue remodeling, that is mediated in large part by interacting molecular signals, primarily cytokines (Xu et al., 2013). Clearance of debris, foreign agents, and/or infectious organisms promotes resolution of inflammation, apoptosis, and the ensuing repair response that encompasses overlapping events involved in granulation tissue, angiogenesis, and re-epithelialization (Rodero and Khosrotehrani, 2010). Within hours, epithelial cells begin to proliferate, migrate, and cover the exposed area to restore functional integrity of the tissue (Xu et al., 2013). TLR4 plays a critical role in pathogen recognition, activation of innate immunity (Lynn et al., 2003), early inflammatory response after traumatic injury (Ozinsky et al., 2000; Huebener and Schwabe, 2013; Keestra et al., 2013), tissue repair, and regeneration (Suga et al., 2014). During wound healing, the activation of TLR by their ligands leads to initiation of signaling cascades, which results in expression of proinflammatory cytokines, chemokines, adhesion molecules, antimicrobial peptides, and proteolytic enzymes that all take part in the complex wound-healing process, especially during the inflammatory stage (Eslani et al., 2014).

Proinflammatory cytokines, such as IL-1β, IL-6, IL12, and TNFα, are often elevated shortly after wounding in both human wounds and animal wound models (Marincola, 2003). Some proinflammatory cytokines and chemokines are essential for normal skin wound-healing processes (Martin, 1997). It is critical that TLR4 responses are tightly regulated to prevent excessive inflammatory responses that could cause tissue damage (Eslani et al., 2014). During normal wound healing, the highest levels of TNFα arise anywhere from 12 to 24 h after wounding (Iizuka and Konno, 2011). After completion of the proliferative phase of wound healing, TNFα returns to basal levels (Xu et al., 2013). It has been reported that the injury to the corneal epithelium induced the expression of TLR4, which stimulated the production of inflammatory cytokines, such as TNFα and IL-6, in the injured corneal epithelium (Kostarnoy et al., 2013). In the present study, both TLR4 and TNFα gene expression were continuously increased during lesion development from d 14 to d 30, but remained at similar levels in healing footpads of LWH as in healthy footpads of NEG. IL-1β mRNA levels were also increased in footpads with lesions on d 14 and reduced in healing footpads on d 27. The five-fold increase of TLR4 in footpads with most severe lesions (d 30) indicates the activation of immune response to fight against pathogens that attack footpads, which leads to inflammatory response and production of inflammatory cytokines, such as TNFα and IL-1β. The prolonged increase of TNFα and IL-1β in lesioned footpads in POS treatment suggests that inflammation was excessively and intensely occurring on footpads with lesions, which impaired wound healing, leading to tissue damage. This is consistent with the impaired wound healing of diabetic foot ulcers by diabetes-enhanced elevated or prolonged expression of TNFα in diabetic patients (Xu et al., 2013). In contrast, the decrease of TNFα, IL-1β, and TLR4 in LWH treatment suggests inflammation and immune activation was diminished upon healing of footpad lesions.

Angiogenesis, the growth of new blood vessels from preexisting vessels, is an important aspect of cell-proliferation phase during wound healing process. This process leads to a temporary increase in the number of blood vessels at the site of injury, which provides oxygen and nutrients to support the growth and function of cells involved in wound healing (Jokinen et al., 2004). VEGF is one of the most potent proangiogenic growth factors in skin that significantly impacts wound healing. Appropriate levels of VEGF are needed for efficient wound repair (Frank et al., 1995; Johnson and Wilgus, 2014). The expression of VEGF was numerically increased in mild-lesioned footpads in both POS and LWH treatment on d 14, suggesting the demand of angiogenesis in response to skin damage on footpads. With development of more severe footpad lesions, the gene expression of VEGF was continuously increased and much greater than healthy footpads (NEG), indicating the increased demand of angiogenesis to repair the wound. However, in healing footpads (LWH), VEGF expression was lower than in severe lesioned footpads (POS), indicating less demand of angiogenesis in healing footpads. These results suggest the gene expression of VEGF indicates the need of angiogenesis, which could be used as a valid biomarker for lesion development and wound-healing process in footpads of broilers.

In summary, direct exposure of chicken feet to excreta by including paper sheets in battery cages induced very high incidence of moderate footpad lesions in 13 d. Removal of paper sheets allowed footpad lesions to almost completely heal in 16 d. Regression analyses indicated the total amount of collagen protein and mRNA levels of TNX, TNC, COL1A1, COL3A1, TIMP3, ITGA2, ITGB1, TNFα, TLR4, VEGF, and the ratio of MMP2 to TIMP were all quadratically associated with footpad lesion scores. It indicates that these parameters are interrelated with dynamic changes of footpad lesion scores. Therefore, they may be used as potential biomarkers for footpad lesion development and wound healing process. The evaluation of FPD has been focused on subjective scoring of lesions present on the plantar surface of footpad. The identification of these biomarkers for footpad lesion development and wound healing may be used to better understand the pathology and etiology of FPD, and find strategies to intervene or prevent the development of footpad lesions and promote the wound-healing process.

## Author contributions

JC and JE designed experiments and wrote manuscript; GT wrote manuscript.

## Funding

This study was funded by Novus International Inc.

### Conflict of interest statement

The authors declare that the research was conducted in the absence of any commercial or financial relationships that could be construed as a potential conflict of interest.

## Abbreviations

FPD: footpad dermatitis
NEG: negative control
POS: positive control
LWH: lesion wound healing
Hyp: Hydroxyproline
SE: standard error
qRT-PCR: quantitative real time polymerase chain reaction
TNX: tenascin X
COL1A1: type I alpha 1 collagen
COL3A1: type III alpha 1 collagen
TIMP3: tissue inhibitor of metalloproteinase 3
TNC: tenascin C
TNFα: tumor necrosis factor alpha
TLR4: Toll-like receptor 4
VEGF: vascular endothelial growth factor
ECM: extracellular matrix.
